# Disease similarity network analysis of Autism Spectrum Disorder and comorbid brain disorders

**DOI:** 10.3389/fnmol.2022.932305

**Published:** 2022-08-18

**Authors:** Joana Vilela, Hugo Martiniano, Ana Rita Marques, João Xavier Santos, Célia Rasga, Guiomar Oliveira, Astrid Moura Vicente

**Affiliations:** ^1^Departamento de Promoção da Saúde e Doenças Não Transmissíveis, Instituto Nacional de Saúde Doutor Ricardo Jorge, Lisbon, Portugal; ^2^Faculty of Sciences, BioISI-Biosystems & Integrative Sciences Institute, University of Lisboa, Lisbon, Portugal; ^3^Unidade de Neurodesenvolvimento e Autismo, Serviço do Centro de Desenvolvimento da Criança, Centro de Investigação e Formação Clínica, Hospital Pediátrico, Centro Hospitalar e Universitário de Coimbra, Coimbra, Portugal; ^4^Faculty of Medicine, University Clinic of Pediatrics and Coimbra Institute for Biomedical Imaging and Translational Research, University of Coimbra, Coimbra, Portugal

**Keywords:** Autism Spectrum Disorder (ASD), Psychiatric genetics, cross-disorder genetics, brain disorders, disease similarity, network analysis, disease community, *de novo* mutations

## Abstract

Autism Spectrum Disorder (ASD) is a neurodevelopmental disorder with heterogeneous clinical presentation, variable severity, and multiple comorbidities. A complex underlying genetic architecture matches the clinical heterogeneity, and evidence indicates that several co-occurring brain disorders share a genetic component with ASD. In this study, we established a genetic similarity disease network approach to explore the shared genetics between ASD and frequent comorbid brain diseases (and subtypes), namely Intellectual Disability, Attention-Deficit/Hyperactivity Disorder, and Epilepsy, as well as other rarely co-occurring neuropsychiatric conditions in the Schizophrenia and Bipolar Disease spectrum. Using sets of disease-associated genes curated by the DisGeNET database, disease genetic similarity was estimated from the Jaccard coefficient between disease pairs, and the Leiden detection algorithm was used to identify network disease communities and define shared biological pathways. We identified a heterogeneous brain disease community that is genetically more similar to ASD, and that includes Epilepsy, Bipolar Disorder, Attention-Deficit/Hyperactivity Disorder combined type, and some disorders in the Schizophrenia Spectrum. To identify loss-of-function rare *de novo* variants within shared genes underlying the disease communities, we analyzed a large ASD whole-genome sequencing dataset, showing that ASD shares genes with multiple brain disorders from other, less genetically similar, communities. Some genes (e.g., *SHANK3, ASH1L, SCN2A, CHD2*, and *MECP2*) were previously implicated in ASD and these disorders. This approach enabled further clarification of genetic sharing between ASD and brain disorders, with a finer granularity in disease classification and multi-level evidence from DisGeNET. Understanding genetic sharing across disorders has important implications for disease nosology, pathophysiology, and personalized treatment.

## Introduction

Autism Spectrum Disorder (ASD) is a neurodevelopmental brain disorder characterized by communication deficits and repetitive behavioral patterns (American Psychiatric Association, 2013). ASD presents as a clinical spectrum, with patients exhibiting variable degrees of clinical severity, compounded by various comorbidities. No single neurobiology underlies ASD, as many molecular mechanisms and biological processes have previously been implicated and are likely reflected in the spectrum of clinical phenotypes observed in patients. ASD segregates in families and a complex genetic architecture is clear, with estimates of up to 1,000 genes potentially implicated (Ramaswami and Geschwind, 2018). While high-risk rare variants in many different genes can be identified in up to 40% of cases, a substantial proportion of ASD risk variance is attributed to common genetic variants with small risk effects (Gaugler et al., 2014), and is still not clearly determined.

A range of brain diseases can co-occur with ASD, which frequently share traits or symptoms (White et al., 2020). However, the combination of comorbid conditions can vary largely between patients, possibly due to different combinations of genetic risk factors. Multiple studies have addressed the genetic overlap between ASD and neuropsychiatric and neurologic conditions, mostly focusing on common genetic variants, uncovering intriguing evidence, particularly for Attention-Deficit/Hyperactivity Disorder (ADHD), Epilepsy, and Schizophrenia (SCZ). Common rare genetic variants have also been identified for Intellectual Disability (ID), one of the most common ASD comorbidities and its subtypes.

For instance, the exome sequencing analysis of approximately 8,000 ASD children, with or without ADHD, discovered a similar burden of rare protein-truncating variants in very conserved genes in both diseases (Satterstrom et al., 2019). ASD frequently co-occurs with ADHD and Epilepsy in children, with rare damaging chromosomal alterations combined with the exposure to environmental factors possibly contributing to the risk of co-occurrence of ASD, ADHD, and Epilepsy (Lo-Castro and Curatolo, 2014). Evidence suggests that ASD and Epilepsy have significant genetic overlap (Novarino et al., 2013). Interestingly, children who have an older sibling diagnosed with ASD have a 70% more chance to have Epilepsy than controls (Christensen et al., 2016).

A Genome-Wide Association Study (GWAS) of over 16,000 ASD cases identified significant genetic overlap between ASD and SCZ (Autism Spectrum Disorders Working Group of The Psychiatric Genomics Consortium, 2017). A common variant genetic overlap between ASD and SCZ was also detected by heritability and correlation analysis based on GWAS studies in very large datasets (Anttila et al., 2018). *Loci* such as 10q24.32 and genes implicated in ASD are also associated with SCZ (Autism Spectrum Disorders Working Group of The Psychiatric Genomics Consortium, 2017). Interestingly, these disorders share a genetic component associated with impairments in social communication; however, they exhibit distinct developmental profiles which are consistent with the different onset of clinical symptoms (St Pourcain et al., 2018). Exome sequencing of 57 trios with sporadic or familial SCZ identified a high proportion of nonsense *de novo* mutations (McCarthy et al., 2014), several in genes implicated in ASD (e.g., *AUTS2, CDH8, MECP2*) and ID (e.g., *HUWE1* and *TRAPPC9*), supporting a shared genetic etiology between these disorders. Exome sequencing of familial Bipolar Disorder (BP) also suggests an overlap with rare genetic variations implicated in SCZ and ASD (Goes et al., 2016). This study identified 84 rare damaging variants that segregated with BP in genes, which were previously reported to have *de novo* variants in ASD and SCZ.

Analysis of sequencing data in large datasets shows that ASD and ID share multiple genes (Satterstrom et al., 2020). This overlap is more evident in syndromes in which patients have both ASD and ID, such as Phelan-McDermid syndrome. This syndrome implicates structural alterations in a chromosomic region that contains the gene *SHANK3* that is strongly associated with ID and ASD. Evidence suggests that mutations in this gene occur in about 1.7% of patients with ID, 0.5% of individuals with ASD, and up to 2% of people with ASD who also have moderate to profound ID (Soorya et al., 2018).

Individuals diagnosed with ASD are two times more likely than control subjects to be diagnosed with Obsessive-Compulsive Disorder (OCD), according to a longitudinal study of nearly 3.4 million people in Denmark over 18 years (Meier et al., 2015). A combined GWAS study of ASD and OCD identified a significant polygenic component of ASD, predicting 0.11% of the phenotypic variance in an independent OCD dataset (Guo et al., 2017).

Given the previous evidence for shared genetic influences between ASD and several comorbid brain disorders, in this study, we sought to further explore the genetic similarity with ASD across a range of brain disorders, using multi-level evidence from well-curated databases and a machine learning approach. Appreciating these genetic overlaps has important implications for disease diagnostic classifications, for understanding the underlying pathophysiology, and for the eventual development of personalized treatment approaches. The specific objectives of the present study were (1) to infer the genetic similarity between ASD and other brain disorders co-occurring with ASD, including disease subtypes; (2) to identify the communities of diseases that have higher genetic similarity with ASD; and (3) to identify likely pathogenic mutations in a large ASD sequencing dataset that may explain the genetic similarities conferred by rare variants across ASD and comorbid brain disorders.

## Methods

The overall methodological process followed in this study is represented in Supplementary File 1.

### Selection of diseases in the DisGeNET database

In this step, we used the DisGeNET database as the data source for disease-disease associations and gene-disease associations (Supplementary File 1). The DisGeNET database includes genetic data on the full spectrum of human diseases as well as normal and abnormal traits (Piñero et al., 2015, 2017, 2020). This database integrates data of human gene-disease associations from several repositories including single gene, complex multigenic, and environmental diseases, integrated by gene and disease vocabulary mapping. We analyzed the DisGeNET dataset of disease-disease associations based on previously implicated genes (file *disease_to_disease_ALL.tsv.gz* from the DisGeNET website^1^), and performed analyses using packages implemented in R studio 2022.02.0 Build 443, R version 4.1.2.^2^ For each pair of diseases, DisGeNET provides the Jaccard Index of disease similarity, which assesses the fraction of shared genes among the diseases according to the proportion of the number of shared genes by the total number of genes implicated in both diseases.

From the DisGeNET file, we selected the disease terms related to ASD and 28 co-occurring psychiatric disorders (White et al., 2020), shown in Table 1. We also selected DisGeNET terms related to Epilepsy, as this neurologic disorder frequently co-occurs with ASD and other psychiatric disorders. Correspondence between the Unified Medical Language System (UMLS) nomenclature used in DisGeNET and the Diagnostic and Statistical Manual of Mental Disorders, Fifth Edition, Text Revision (DSM-V; American Psychiatric Association, 2013), was done using the codes from the International Classification of Diseases ICD-10/ICD-10-CM, which belong to the medical coding system designed by the World Health Organization (World Health Organization, 2004).

**TABLE 1:** Disease terms selected according to the International Classification of Diseases ICD-10/ICD-10-CM codes (the latest release, ICD-11, is not yet available in DisGeNET), the medical coding system designed by the World Health Organization to catalog health conditions by categories of similar diseases under which more specific conditions are listed. All the disease categories were obtained from the DSM-5, except Epilepsy, which is a neurologic disorder.

ICD-10/ICD-10-CM code	Unified Medical Language System (UMLS) nomenclature	DSM-5 disease category
F23; F23.9	Acute transient psychotic disorder	Schizophrenia Spectrum Disorder and other psychotic disorders
F90; F90.9	Attention-Deficit/Hyperactivity Disorder	Neurodevelopmental disorders
F90.2	Attention-Deficit/Hyperactivity Disorder, combined type	Neurodevelopmental disorders
F84.0	Autism Spectrum Disorders (the term Autistic Disorder is not in use in DSM-5)	Neurodevelopmental disorders
F31; F31.9	Bipolar Disorder	Bipolar and related disorders
F31.81	Bipolar II Disorder	Bipolar and related disorders
F06.1	Catatonia, Organic*	Occurs in the context of disorders from different disease categories*
F06.1	Catatonia**	Occurs in the context of disorders from different disease categories**
F34.0	Cyclothymic Disorder	Bipolar and related disorders
F22.0	Delusional disorder	Schizophrenia Spectrum Disorder and other psychotic disorders
G40; G40.9; G40.909	Epilepsy	–
F90.1	Hyperkinetic conduct disorder	Neurodevelopmental disorders
F21	Incipient Schizophrenia	Schizophrenia Spectrum Disorder and other psychotic disorders/Personality Disorders
F22	Involutional paraphrenia	Schizophrenia Spectrum Disorder and other psychotic disorders
F79	Intellectual Disability (substitutes the term Mental deficiency, not in use in DSM-5)***	Neurodevelopmental disorders
F79	Intellectual Disability (substitutes the term Mental handicap, not in use in DSM-5)***	Neurodevelopmental disorders
F70-F79.9	Intellectual Disability (substitutes the term Mental Retardation, not in use in DSM-5)***	Neurodevelopmental disorders
F79	Intellectual Disability (substitutes the term Mental Retardation, not in use in DSM-5)***	Neurodevelopmental disorders
F70	Mild Intellectual Disability (substitutes the term Mild Mental Retardation, not in use in DSM-5)***	Neurodevelopmental disorders
F71	Moderate Intellectual Disability	Neurodevelopmental disorders
F29	Nonorganic psychosis	Schizophrenia Spectrum Disorder and other psychotic disorders
F42; F42.9; F42.8	Obsessive-Compulsive Disorder	Obsessive-compulsive and related disorders
F22	Paranoia	Schizophrenia Spectrum Disorder and other psychotic disorders
F73	Profound Intellectual Disability (the term Profound Mental Retardation is not in use in DSM-5)***	Neurodevelopmental disorders
F23	Psychosis, Brief Reactive	Schizophrenia Spectrum Disorder and other psychotic disorders
F25.0	Schizoaffective disorder, bipolar type	Schizophrenia Spectrum Disorder and other psychotic disorders
F20.81	Schizophreniform Disorders	Schizophrenia Spectrum Disorder and other psychotic disorders
F20.81	Schizophreniform psychosis NOS	Schizophrenia Spectrum Disorder and other psychotic disorders
F21	Schizotypal Personality Disorder	Schizophrenia Spectrum Disorder and other psychotic disorders/Personality Disorders
F72	Severe Intellectual Disability	Neurodevelopmental disorders

Correspondence between the Unified Medical Language System (UMLS) nomenclature used in DisGeNET and DSM-5 was done by the ICD-10/ICD-10-CM codes.

*The nosology used in DisGeNET with respect to Catatonia, follows a classification that was present in the previous version of the Diagnostic and Statistical Manual of Mental Disorders (DSM-IV), which defined that Organic Catatonia was related to the occurrence of Catatonia due to a General Medical Condition, clearly distinct from a Schizophrenia subtype.

**The Catatonia here is not the Organic Catatonia but includes Catatonia present in psychotic and mood disorders, which includes the catatonic subtype of Schizophrenia from the DSM-IV (Tandon et al., 2013). The catatonic subtype of Schizophrenia from the DSM-IV will be deleted in the DSM-5 revision along with other Schizophrenia subtypes (Tandon and Carpenter, 2012), and Catatonia is currently a specifier for Schizophrenia.

***Intellectual Disability is the term used in this study in substitution to the DisGeNET terms Mental deficiency, Mental handicap, and Mental retardation as these terms are no longer in use in DSM-5.

### Construction of a network of disease gene-based similarities between Autism Spectrum Disorder and comorbid brain disorders and disease community detection

The input data source for this analysis is the filtered DisGeNET file of disease-disease associations from the previous step (Supplementary File 1). The network of disease-disease similarities was produced using the DisGeNET Jaccard Index, which calculates the genetic similarity for each disease pair, as the weight of the edge connecting each pair of diseases. The network was generated using the igraph R package.^3^ Network disease communities were identified using the Leiden community detection algorithm (Traag et al., 2019) implemented in the R package LeidenAlg (Kharchenko et al., 2022). This algorithm identifies network communities based on modularity optimization. Modularity compares the number of connections inside a community with the expected number of connections for that community in a random network with the same number of nodes and keeping the same degree (number of connections of a node with other nodes). Using this method, we constructed a network of disease-disease similarities and calculated the number of disease communities optimizing the modularity parameter. Network edition and visualization were done with Cytoscape v 3.7.2 (Shannon et al., 2003).

### Analysis of gene sets underlying disease communities, and pathway and molecular function enrichment analysis

In this step, we identified several gene sets: (1) genes shared within each community; (2) genes that overlap among different disease communities; and (3) genes that are exclusive from each community (Supplementary File 1). After performing disease community detection, we analyzed the DisGeNET dataset *all_gene_disease_associations.tsv.gz* available at https://www.disgenet.org/downloads to identify the gene sets. This dataset contains all gene-disease associations in the database.

Pathway and molecular function enrichment used the list of genes that are exclusive to each disease community and was performed using Reactome pathways (Jassal et al., 2020) and Gene Ontology (GO; Carbon et al., 2009) molecular functions, using g:Profiler (Raudvere et al., 2019; Supplementary File 1). The Gene Ontology (GO) resource^4^ develops structured controlled ontologies to characterize genes and their products. The GO Consortium has developed AmiGO, a web-based application that allows users to search, sort, analyze, visualize, and download the data of interest. Reactome^5^ is a manually curated and peer-reviewed pathway database that provides bioinformatic tools for the visualization, interpretation, and analysis of pathway information with applications in genome analysis, modeling, and systems biology. Only results below a threshold of 0.05 for the False Discovery Rate were considered.

### Identification of rare *de novo* loss of function Single Nucleotide Variants within genes from network disease communities, in Autism Spectrum Disorder patients

To validate the prediction that ASD patients have mutations in genes shared among comorbid brain disorders, we searched for Single Nucleotide Variants (SNVs), namely *de novo* loss of function (LoF) mutations, in an ASD Whole Genome Sequencing (WGS) public dataset. We focused on rare *de novo* mutations because this class of variants plays an important role in neurodevelopmental diseases, including ASD, ID, ADHD, and others. For this purpose, we searched the large dataset of genomic variants from WGS of ASD subjects available in the MSSNG database^6^ (Yuen et al., 2017; Supplementary File 1). MSSNG is a collaboration between the ASD organization Autism Speaks,^7^ Google, and the research community to create the world’s largest genomic database on ASD. MSSNG seeks to make data available from the WGS of 10,000 individuals from ASD families. The omitted letters in MSSNG (pronounced “missing”) represent the missing information about ASD that the project seeks to deliver (Yuen et al., 2017).

The dataset was already filtered for quality parameters (Yuen et al., 2017), namely: (1) variants with genotype quality of at least 99 (GQ for Illumina; VAF for Complete Genomics); (2) variants with Minor Allele Frequency (MAF) below 1%; and (3) variant call more than 95% of the time as a reference allele and less than 1% of the time as a variant in the parents. We performed a systematic analysis of data of 3,881 ASD individuals. For the selection of rare *de novo* predicted LoF SNVs, we inspected the variants with predicted pathogenic effect in protein function or structure, according to the Ensembl Variant Effect Predictor (VEP; McLaren et al., 2016), and only LoF variants of frameshift, stop gain and stop loss, were present in the dataset. Finally, we identified the proportion of rare *de novo* LoF SNVs present in genes from each disease community of the network, identified genes with variants in all disease communities, and characterized the genes from the ASD disease community (Supplementary File 1).

Genes from the ASD disease community with more than one rare *de novo* LoF SNV were also characterized according to the Simons Foundation Autism Research Initiative (SFARI) gene scoring module.^8^ SFARI is an ASD-dedicated database that incorporates a gene scoring module to establish a gene rank according to the strength of the evidence that associates a given gene with ASD. The Syndromic category (Category S) includes mutations that are associated with a substantial degree of increased risk and consistently linked to additional traits not required for an ASD diagnosis. If there is independent evidence implicating a gene in idiopathic ASD, it is listed as ‘‘#S’’ (e.g., 1S, 2S). If there is no independent evidence, the gene is listed only as ‘‘S.’’ Category 1 genes have been clearly implicated in ASD (presence of at least three *de novo* likely-gene-disrupting mutations) and are also found in the gene list of a large ASD research study, SPARK^9^ (SPARK Consortium, 2018), or on the list of genes reported by Satterstrom et al. (2020). All the genes in this category reach a False Discovery Rate threshold of < 0.1 (threshold used in Satterstrom et al. (2020) for the identification of ASD risk genes in a large exome sequencing ASD cohort), and some reach the threshold of genome-wide significance. Genes from the SFARI 3 category were not used, as they constitute weaker ASD candidates.

## Results

### Construction of a network of gene-based disease similarities between Autism Spectrum Disorder and comorbid brain disorders and disease community detection

**FIGURE 1:**
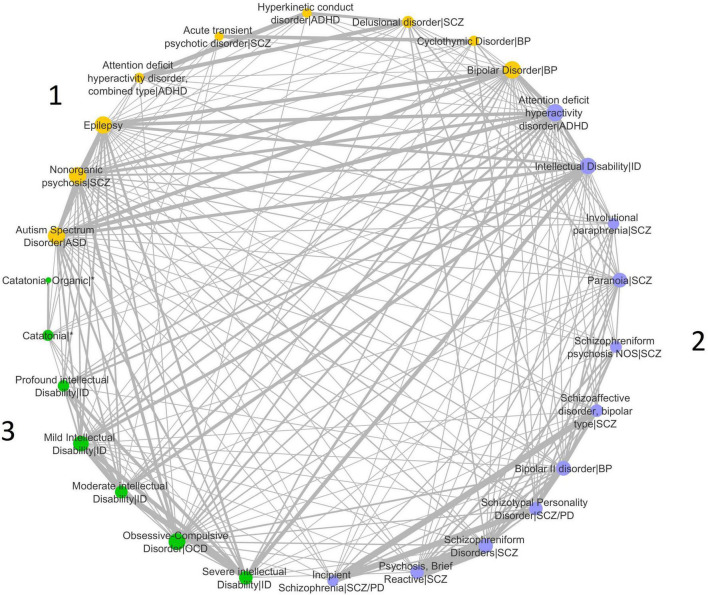
Disease similarity network. The implementation of the Leiden algorithm for the identification of disease communities in the network identified three disease communities. The different communities are indicated with different colors in the network, and the diseases included in each community are shown in the figure. Network nodes (or vertices) represent diseases. Edges (or links) represent disease-disease associations. Edge thickness is proportional to weight and represents the degree of similarity between disease pairs computed by the Jaccard index. Circles sizes reflect the node degree (Supplementary Table 1), which represents the number of connections the node has to other network nodes. ASD, Autism Spectrum Disorder; ADHD, Attention-Deficit/Hyperactivity Disorder; SCZ, Disease included under the Schizophrenia Spectrum Disorder and other psychotic disorders diagnosis; ID, Disease included under the Intellectual Disability diagnosis; BP, Disease included in the Bipolar and related disorders diagnosis; SCZ/PD, Disease included under the Personality Disorder diagnosis and under the Schizophrenia Spectrum Disorder and other psychotic disorders diagnosis; OCD, Disease included under the Obsessive-Compulsive and related disorders diagnosis; *Occurs in the context of disorders from different disease categories. Duplicate nodes in the same community were omitted from the figure but used in the analyses: for ASD—nodes from obsolete disease terms that are not used in the DSM-5 edition; for Intellectual Disability—nodes from disease terms that are not present in the DSM-5 edition are substituted for updated DSM-5 terms.

We analyzed 309 disease-disease associations based on the genetic similarity of a set of 31 brain disease terms from the DisGeNET dataset of disease-disease associations (Table 1). We constructed a network of disease-disease similarities and calculated the number of disease communities optimizing the modularity parameter of the Leiden algorithm (Figure 1). The highest modularity positive score for the dataset was 0.06, indicating that the number of connections within groups exceeds the number of expected connections by chance.

Three disease communities were identified (Figure 1). ASD is placed in Community 1 with Epilepsy, ADHD combined type, Hyperkinetic conduct disorder (HC), and some of the diseases included in the SCZ Spectrum, Bipolar Disorder (BP), and Cyclothymic Disorder (CD). Community 2 integrates the majority of the diseases in the SCZ spectrum, as well as ADHD, BP type II disorder, and Intellectual Disability (ID). Subtypes of ID are also present in Community 3, along with Obsessive-Compulsive Disorder (OCD) and Catatonia. Network connectivity for each disease is measured by the node degree. ASD, BP, Epilepsy, and Nonorganic psychosis (NP) in Community 1 are the diseases with the highest node degree, indicative of the largest number of connections to other diseases in the entire network. The strength of disease connections is represented by line widths, as thicker lines represent stronger connections. The strongest ASD intra-community connections are with Epilepsy and BP. ASD also shows very strong connections to ADHD and ID in Community 2. A strong connection is also found between ASD and BP in Community 1. ADHD and ID are the diseases with the highest node degree in Community 2, while OCD, Mild ID, and severe ID in Community 3 show the highest number of connections to other diseases (Figure 1 and Supplementary Table 1). ID subtypes are mostly clustered in Community 3, and we can observe a strong connection between ID (in Community 2) and most of these subtypes, independently of severity level. Strong connections are also detected between ADHD and Delusional disorder/SCZ, and Epilepsy and BP.

### Analysis of gene sets underlying disease communities, and pathway and molecular function enrichment analyses

Community 1 has the largest number of genes (3,191 genes), while Community 2 includes a total of 1,276 genes, and Community 3 has 1,045 genes (Supplementary File 2). The three gene lists partially overlap (Supplementary File 2). However, some of the genes implicated in the disease-disease associations analyzed are exclusive from each community. The proportion of the number of exclusive genes by the total number of genes for each community is 67.63% for Community 1, 30.49% for Community 2, and 39.90% for Community 3 (Supplementary File 2).

For the pathway enrichment analyses, we focused on the list of exclusive genes from each disease community and, for all three gene lists, calculated the enrichment in GO molecular functions (GO:MF) and Reactome pathways (REAC) (Supplementary File 3). Some of these pathways were previously associated with disorders in each of these communities. The top five enriched terms for each community are shown in Table 2.

**TABLE 2:** Top five Gene Ontology molecular functions and Reactome pathways enrichment for communities’ exclusive genes.

Community 1			
**Source**	**Term_name**	**Term_id**	**Adjusted_*p*_value**
REAC	Signal Transduction	REAC:R-HSA-162582	1.95E-12
REAC	Neuronal System	REAC:R-HSA-112316	1.31E-09
REAC	Signaling by GPCR	REAC:R-HSA-372790	4.57E-08
REAC	GPCR downstream signalling	REAC:R-HSA-388396	4.91E-08
REAC	Signaling by Nuclear Receptors	REAC:R-HSA-9006931	3.81E-07
GO:MF	signaling receptor binding	GO:0005102	7.68E-19
GO:MF	enzyme binding	GO:0019899	5.55E-16
GO:MF	ion binding	GO:0043167	6.16E-15
GO:MF	ion transmembrane transporter activity	GO:0015075	1.64E-14
GO:MF	signaling receptor activity	GO:0038023	5.67E-13

**Community 2**			

**Source**	**Term_name**	**Term_id**	**Adjusted_*p*_value**

REAC	Diseases of Mismatch Repair (MMR)	REAC:R-HSA-5423599	1.20E-03
REAC	RHOQ GTPase cycle	REAC:R-HSA-9013406	2.31E-02
REAC	Diseases of DNA repair	REAC:R-HSA-9675135	2.36E-02
REAC	RHOJ GTPase cycle	REAC:R-HSA-9013409	3.04E-02
REAC	RAC3 GTPase cycle	REAC:R-HSA-9013423	3.04E-02
GO:MF	single base insertion or deletion binding	GO:0032138	1.71E-02
GO:MF	mismatch repair complex binding	GO:0032404	1.71E-02
GO:MF	DNA insertion or deletion binding	GO:0032135	3.87E-02

**Community 3**			

**Source**	**Term_name**	**Term_id**	**Adjusted_*p*_value**

REAC	Respiratory electron transport. ATP synthesis by chemiosmotic coupling and heat production by uncoupling proteins.	REAC:R-HSA-163200	1.18E-04
REAC	Complex I biogenesis	REAC:R-HSA-6799198	1.18E-04
REAC	Respiratory electron transport	REAC:R-HSA-611105	1.18E-04
REAC	The citric acid (TCA) cycle and respiratory electron transport	REAC:R-HSA-1428517	1.18E-04
REAC	Post-translational modification: synthesis of GPI-anchored proteins	REAC:R-HSA-163125	9.86E-03
GO:MF	catalytic activity	GO:0003824	1.44E-07
GO:MF	oxidoreduction-driven active transmembrane transporter activity	GO:0015453	4.97E-06
GO:MF	NADH dehydrogenase (ubiquinone) activity	GO:0008137	4.97E-06
GO:MF	NADH dehydrogenase (quinone) activity	GO:0050136	4.97E-06
GO:MF	NADH dehydrogenase activity	GO:0003954	5.08E-06

### Identification of rare *de novo* loss of function Single Nucleotide Variants in Autism Spectrum Disorder datasets

We analyzed 3,881 ASD cases from the MSSNG whole genome sequencing dataset and identified 366 rare (MAF < 1%) *de novo* LoF SNVs, within 336 genes (Yuen et al., 2017; Supplementary Table 2). The majority of these variants (43.32%) fall in the predicted effect category of *stop gain* variants, followed by *frameshift deletions* (36.51%) and *frameshift insertions* (17.71%; Supplementary File 4).

We further searched this ASD dataset specifically for rare *de novo* LoF SNVs within the genes associated with each disease community of the network. As expected, we found that Community 1 diseases share the largest proportion (33.61%) of rare *de novo* LoF SNVs, followed by Community 3 (22.68%) and Community 2 (18.85%; Supplementary File 5).

**FIGURE 2:**
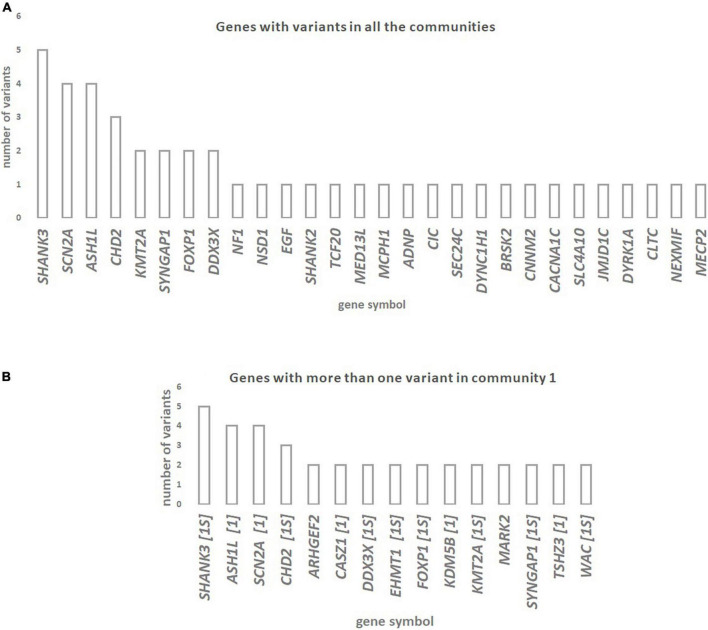
**(A)** Genes with rare *de novo* LoF SNVs in ASD patients are shared across diseases from the three communities of the network of disease-disease similarities. **(B)** Genes with more than one rare *de novo* LoF SNV in ASD patients that are shared across Community 1 diseases (which includes ASD) of the network of disease-disease similarities. Whenever genes are also in the SFARI gene scoring module as candidate genes for ASD (https://gene.sfari.org/database/gene-scoring/), we display the evidence score next to the gene symbols.

The genes with rare *de novo* LoF SNVs that are shared among all communities are shown in Figure 2A. The genes *SHANK3, ASH1L, SCN2A*, and *CHD2* have the highest number of rare *de novo* LoF SNVs (three to five variants) in ASD patients. The genes with more than one rare *de novo* LoF SNV in Community 1 are shown in Figure 2B. Genes that are also in the SFARI gene scoring module as candidate genes for ASD are shown. Most of the 15 genes shared by Community 1 diseases with more than one variant are included in the SFARI ASD list of candidate genes in high evidence categories, namely category 1 (five genes) and category 1S (eight genes). Genes with more than one rare *de novo* LoF SNV in communities 2 and 3 are shown in Supplementary File 5. A summary of the results of this study can be found in Supplementary Table 3.

## Discussion

In this study, we investigated the shared genetic component between ASD and other brain disorders that often co-occur in ASD patients. We hypothesize that the clinical variability that characterizes ASD, and the occurrence of comorbidities, may be partially explained by shared genetic components among these disorders and ASD. To further understand this question, we explored whether some of these disorders are genetically more similar to ASD than others that do not frequently co-occur with this disorder. For this purpose, we built a disease similarity network, using the shared genetic architecture as a means to assess disease-disease similarities.

Using this approach, we showed that ASD is one of the five disorders with more connections in the disease similarity network. This result indicates that ASD indeed shares genes that have been previously implicated in its etiology with many of the 29 co-occurring brain disorders analyzed.

We next used the Leiden community detection algorithm to cluster all diseases into three communities, according to gene-based genetic similarity. ASD is included in Community 1, which encompasses a heterogeneous group of diseases, namely Epilepsy, ADHD subtypes, and some of the conditions that are part of the BD and the SCZ spectra. This community also shows the largest number of shared genes. Most of the disorders of Community 2 are SCZ subtypes, and ID is highly represented in Community 3 and shares a fewer number of genes. The heterogeneous nature of Community 1 suggests that ASD has genetic similarities with a larger number of disorders than SCZ, BP, and ID. This is expected and in accordance with our hypothesis, given that the clinical presentation of ASD is so variable, compounded by multiple comorbidities, and that this variability is frequently attributed to the large number of genes that have been implicated in its etiology (Ramaswami and Geschwind, 2018). It is somewhat unexpected that both ADHD and ID cluster in Community 2, which generally groups other neuropsychiatric diseases with a late adolescent or adult-onset. Furthermore, ID subtypes are mostly clustered in Community 3, and we can observe a strong connection between ID (in Community 2) and most of these subtypes, independently of severity level. This unexpected community formation may reflect some bias in the terminology used by DisGeNET, as ID encompasses all the ID subtypes, and fewer genetic studies may be available for each of the subtypes.

The strongest ASD intra-community connections are with Epilepsy and BD. While Epilepsy is a common comorbidity of ASD, the connection with BD is not frequently reported. ASD also shows very strong connections to two diseases in Community 2, namely ADHD and ID. These two neurodevelopmental disorders are, together with Epilepsy, the most common comorbidities of ASD. The finer nosological granularity provided by this approach may also be revealing previously less clear genetic similarities. For instance, a strong connection is found between ASD and BD in Community 1. It is worth noting that ASD, ADHD, ID, and Epilepsy are normally diagnosed early in life and, as such, most studies are carried out in children. On the other hand, SCZ, psychosis, and BP are later onset diseases. Because few studies are focusing on adults with ASD (Forde et al., 2021), not much clinical evidence is available regarding the co-occurrence of these adult diseases and ASD, but it can be detected using this approach based on gene sharing. Interestingly, the approach also detects strong connections and community grouping between other childhood onset and adult onset brain diseases, for instance, between ADHD and Delusional disorder/SCZ, and Epilepsy and BD. These findings provide evidence for genetic relationships that were previously not considered and suggest that is worth exploring further the comorbidities in datasets of adults diagnosed with childhood-onset disorders.

The community detection algorithm assigns ID and ASD to separate communities. Even though ASD and ID are known to share candidate genes, the results show that there is enough genetic distance to place both disorders in separate communities. This may indicate that ASD patients with ID may have mutations in a subset of genes that are more related to ID than ASD patients without ID. This is in accordance with previous evidence that showed that some ASD-risk genes have higher frequencies of disruptive *de novo* variants in people ascertained for developmental delay (Satterstrom et al., 2020).

Previous work on clustering analysis of psychiatric disorders suggested that ASD and mood and psychotic disorders (which included SCZ and BP) cluster in different groups (Lee et al., 2019). Our results have some degree of overlap with previous evidence as we identify a community composed of most of the SCZ spectrum disorders (Community 2). However, as we have genetic annotations for disease subtypes as input data, we were also able to infer which SCZ-related disorders have more genetic similarity with ASD than others.

Enrichment analysis using genes exclusively from each of the three communities allowed the identification of shared biological pathways and molecular functions among these brain disorders. Community 1 is enriched in signal transduction and neuronal system mechanisms. These pathways have been implicated in some diseases from Community 1, for instance, the *dysfunction in neuronal activity-dependent signaling pathways* in ASD (Ebert and Greenberg, 2013), the *dysregulation in neuronal apoptosis-regulatory pathways* in Epilepsy (Bozzi et al., 2011), or alterations in *G-protein-coupled receptors (GPCRs)* in ASD, SCZ, and BP (Monfared et al., 2021). The top five terms enriched in GO molecular functions and Reactome pathways for genes exclusive of Community 2 are related to DNA repair and mismatch repair. Mismatch repair is a cellular pathway that corrects base mispairs occurring during DNA replication (Graham et al., 2019). High-level DNA damage has been implicated in psychiatric disorders (Raza et al., 2016), and candidate genes suggesting a role for this mechanism in Schizophrenia have been identified (Odemis et al., 2015). Genes exclusive of Community 3 are enriched in terms related to mitochondrial complex 1 NADH dehydrogenase, a finding in accordance with the typical association of Mitochondrial complex I deficiency with severe ID, encephalopathy, ataxia, and dystonia (Loeffen et al., 2000; Distelmaier et al., 2009; Pagniez-Mammeri et al., 2012; Valiente-Pallejà et al., 2018).

We further analyzed rare *de novo* LoF SNVs in 3,881 ASD cases from the MSSNG WGS cohort, to explore whether ASD patients have pathogenic mutations in genes implicated in multiple brain disorders. *de novo* mutations in ASD, and in human disease in general, are maintained at low frequencies in the population by natural selection (Alonso-Gonzalez et al., 2018), and therefore, rare risk alleles tend to be eliminated, while common alleles that are benign or neutral show signs of positive selection (Polimanti and Gelernter, 2017). However, *de novo* mutations can have strong effects on protein structure and/or function and, despite being rare individually, may capture an important component of the heritability for complex genetic diseases (Veltman and Brunner, 2012). Supporting this hypothesis, we identified, in these ASD subjects, rare *de novo* LoF SNVs within genes previously associated with other disorders in all three communities.

We observed that the genes *SHANK3, ASH1L, SCN2A*, and *CHD2*, which are candidate genes for diseases in all three communities, have a higher number of *de novo* rare LoF SNVs in ASD subjects. These genes are strong candidates for ASD, according to the SFARI gene category scoring. These observations suggest that ASD patients have deleterious mutations in genes shared with other brain disorders from the same or different communities. In fact, previous reports implicate these genes in several of the disorders analyzed. For instance, mutations in *SHANK3* (SH3 and Multiple Ankyrin Repeat Domains 3), which encodes a synaptic scaffold protein supporting the organization of hundreds of other synaptic proteins, have been reported in patients with ASD, ID, and SCZ (Durand et al., 2012; Monteiro and Feng, 2017). The *ASH1L* gene (ASH1-Like Histone Lysine Methyltransferase), that encodes a histone methyltransferase involved in chromatin modification, binds to the promoter region of *NRXN1* (Neurexin 1) inhibiting the transcriptional activity of *NRXN1* and dysregulating synapse formation (Zhu, 2016; Zhang et al., 2021). Several reports associate this gene with ID, delayed speech, and seizures (Okamoto et al., 2017). Mutations in the *CHD2* gene (Chromodomain Helicase DNA Binding Protein 2), which encodes a member of the chromodomain helicase DNA-binding (CHD) family of proteins, have been identified in ASD, ID, and Epilepsy (Luo et al., 2022). The present approach was therefore successful in identifying genes underlying the shared genetic component between ASD and the diseases analyzed.

Previous studies have addressed the question of cross-disorder genetic overlap, mainly focusing on common genetic variation resulting from Genome-Wide Association Studies (GWAS; Autism Spectrum Disorders Working Group of The Psychiatric Genomics Consortium, 2017; Anttila et al., 2018; Lee et al., 2019). Instead of addressing common variations shared between disorders, this study complemented the previous evidence by focusing on rare *de novo* variants. Rare *de novo* mutations contribute substantially to an individual’s ASD risk (Iossifov, 2012; Neale, 2012; Genovese and Butler, 2020), and altogether explain ASD in a significant fraction of patients (Sanders, 2012). Furthermore, rare *de novo* mutations are extremely useful to identify biological pathways that can be disrupted by different mutations in multiple genes. Understanding which genes mutated in ASD are shared by co-occurring brain conditions is an important step to understanding the clinical variability of ASD, and eventually to defining targets for therapeutic development.

Our methodological approach improves previous studies in several aspects. First, the data on gene-disease associations comes from a wide range of sources, including curated genetic databases, animal models, or GWAS data, and therefore provides converging evidence from multiple sources. Second, we used gene-disease annotations to several disorders that compose larger disease classes, for instance, multiple levels of ID disease severity, several disorders in the SCZ spectrum, or different ADHD and BP clinical profiles. We, therefore, took advantage of existing fine-grained disease classifications, gaining resolution to explain the architecture of disease subtypes and variable clinical presentations, as we did not assume that the genetic component of a disease is equally distributed across disease subtypes. Third, we characterized the genes implicated in a disease community-driven framework, by analyzing disease pairs in the context of their genetic relationships with several other disorders in the network instead of analyzing pairs of diseases independently. The methodology developed still has some limitations, as the annotation coverage of gene-disease association is uneven among diseases, which can lead to under or overestimated disease similarities. Improved annotation and curation of databases is crucial for approaches based on the analysis of large datasets and needs to be addressed very seriously for medical use of results from artificial intelligence approaches.

Overall, this work provided further evidence for a shared genetic architecture between ASD and several other brain disorders, including some frequent comorbidities of ASD. It also identified genes and biological processes that overlap between brain disease communities and showed that ASD patients present rare *de novo* LoF variants in genes previously associated with frequent comorbid disorders. Future studies on the analysis of disease-disease similarities should be developed with the aim of expanding this analysis to other ASD co-occurring conditions that are not in the scope of the mental illness, in particular, diseases that affect the immune system (Meltzer and van de Water, 2017) and the gastrointestinal tract, and may be related with genetically determined microbiome constitution (Liu et al., 2021).

Importantly, this study showed the value of a data-driven approach, using data from heterogeneous sources to characterize the genetic overlap between diseases and, in particular, to gain insights into the shared genetic architecture of ASD and other brain disorders.

## Data availability statement

ASD whole-genome sequencing dataset analysed annotated_de_novo_variants_20200624.xls was obtained from the MSSNG web-site (https://research.mss.ng/).

## Ethics statement

The studies involving human participants were reviewed and approved by the Institutional Review Boards or Ethical Committees of the participating sites. Written informed consent to participate in this study was provided by the participants’ legal guardian/next of kin.

## Author contributions

JV, HM, and AV developed the concept for this work. Data curation, methodological development, and formal analysis were carried out by JV, with strong support from HM and contributions from JS, AM, CR, and GO. GO and AV provided resources. AV and HM provided overall supervision and coordination. JV wrote the initial draft, revised, and edited by HM and AV. All authors contributed and revised the article, and approved the submitted version.

## Footnotes

1
https://www.disgenet.org/downloads

2
http://www.rstudio.com/

3
https://igraph.org/

4
http://geneontology.org/

5
https://reactome.org/

6
https://research.mss.ng/

7
https://www.autismspeaks.org/

8
https://gene.sfari.org/database/gene-scoring/

9
https://sparkforautism.org/
