# Supplementary figures and images for: Disease similarity network analysis of Autism Spectrum Disorder and comorbid brain disorders
Source: Front Mol Neurosci. 2022 Aug 18;15:932305. doi: 10.3389/fnmol.2022.932305 (PMC9434349; doi:10.3389/fnmol.2022.932305)

**
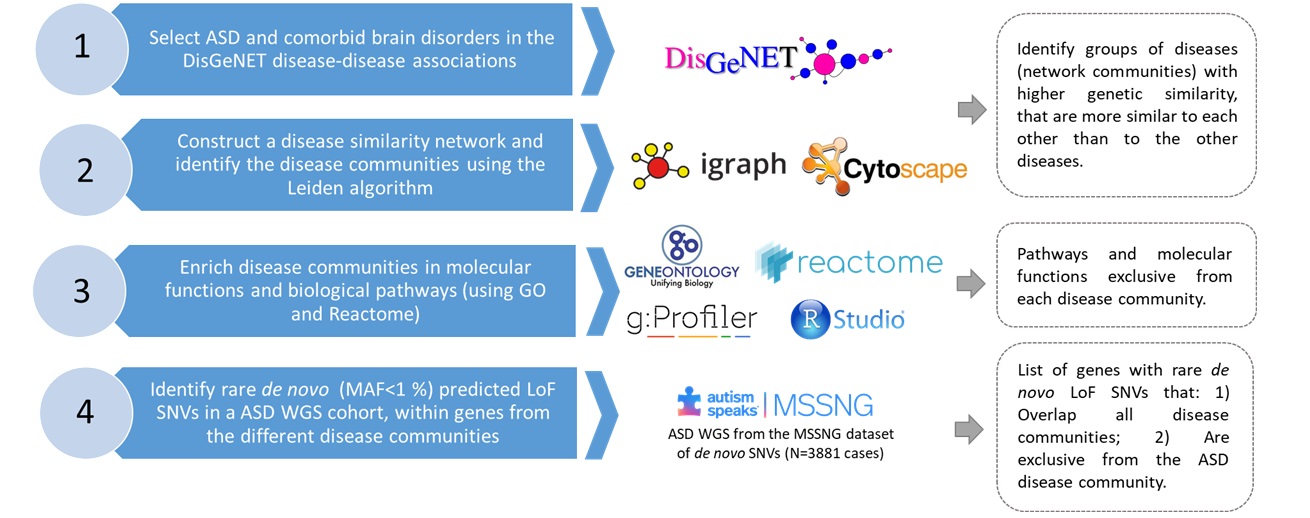
**

**Supplementary File 1.** **Workflow for building and analyzing the disease similarity network.**

Supplement: Supplementary file 1 [file Table_1.DOCX]
