# Supplementary material for: Disease similarity network analysis of Autism Spectrum Disorder and comorbid brain disorders
Source: Front Mol Neurosci. 2022 Aug 18;15:932305. doi: 10.3389/fnmol.2022.932305 (PMC9434349; doi:10.3389/fnmol.2022.932305)
Supplement: Supplementary file 4 [file Table_4.DOCX]

**
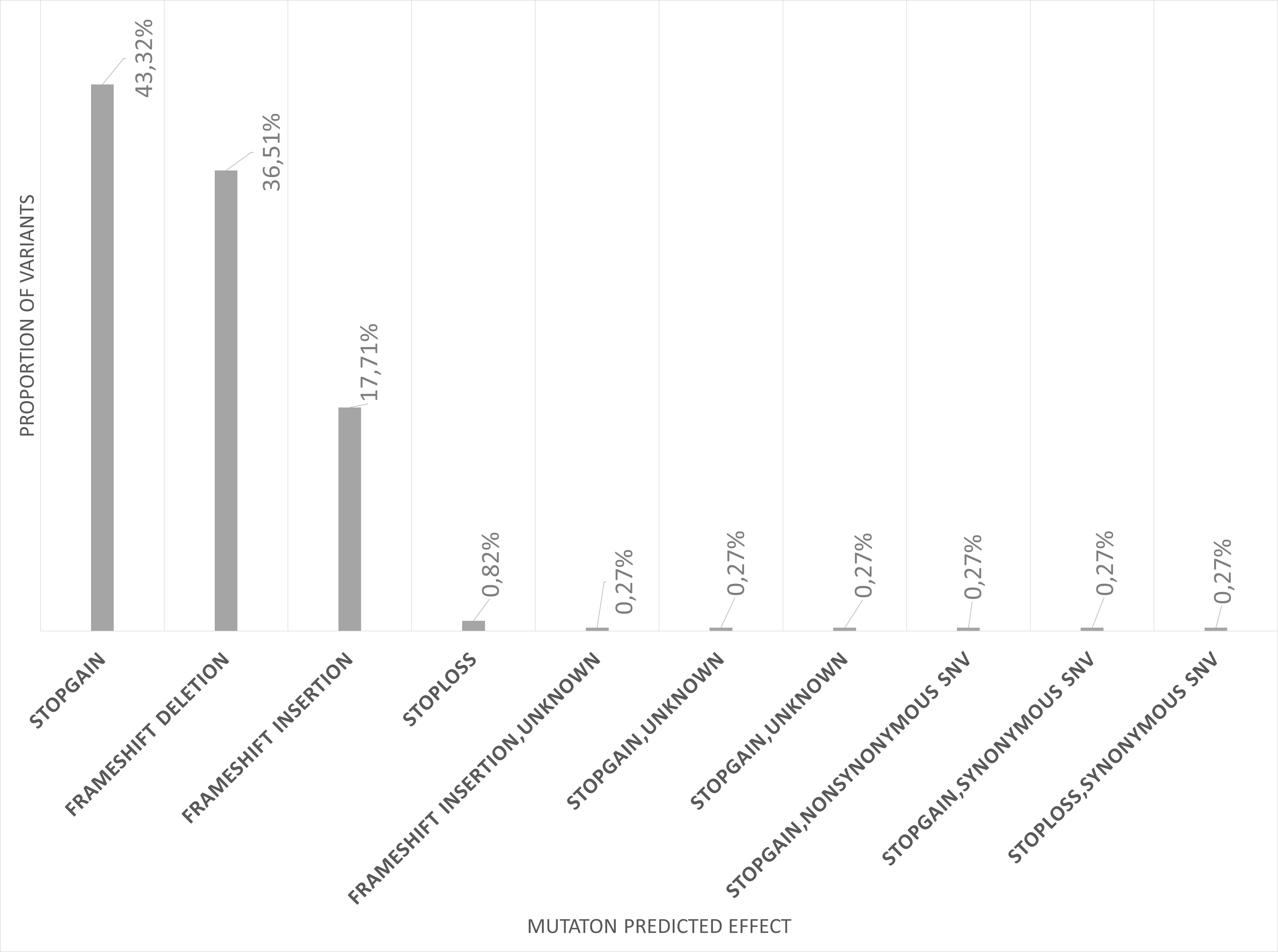
**

**Supplementary File 4.** Distribution of variant effect classes for rare *de novo* LoF SNVs selected in the MSSNG WGS dataset.
